# Electrodes for Semiconductor Gas Sensors

**DOI:** 10.3390/s17040683

**Published:** 2017-03-25

**Authors:** Sung Pil Lee

**Affiliations:** Department of Electronic Engineering, Kyungnam University, 7 Kyungnamdaehak-ro, Masanhappo-gu, Changwon 51767, Korea; sensors@kyungnam.ac.kr

**Keywords:** electrodes, semiconductor gas sensors, electrode materials, interfacial layer, transport mechanism

## Abstract

The electrodes of semiconductor gas sensors are important in characterizing sensors based on their sensitivity, selectivity, reversibility, response time, and long-term stability. The types and materials of electrodes used for semiconductor gas sensors are analyzed. In addition, the effect of interfacial zones and surface states of electrode–semiconductor interfaces on their characteristics is studied. This study describes that the gas interaction mechanism of the electrode–semiconductor interfaces should take into account the interfacial zone, surface states, image force, and tunneling effect.

## 1. Introduction

There is a growing demand for gas sensors for efficient use of energy and raw materials, as well as to reduce environmental pollution despite increasingly complex manufacturing processes. The Taguchi sensor that detects reducing gases is the most well-known gas sensor [1], whereas an oxygen sensor based on an ion-conducting sensor is the second most famous type [2,3,4,5]. Research and development for gas sensors is conducted in two stages. The first stage is to develop a new sensor whose application is empirically optimized. The characteristics of sensitivity, selectivity, long-term drift, and reliability are defined though its operation mechanism, which is often not fully understood at that stage [6,7,8,9,10]. The second stage is to modify, optimize, and standardize the developed sensor system [11,12,13,14]. The developed sensors can measure change or values of current [15,16], impedance [17,18], capacitance [19], frequency [20], potential difference [21,22] and electromotive force [23]. In addition, the correlation between the sensor structure and electrode is very important to expressively depict these phenomenological parameters that characterize the sensor.

Recently, the surface and interface science for semiconductor gas sensors have been extensively studied. In addition, gas sensing mechanisms [24,25,26], gas sensor technology [27], the semiconductor junction for gas sensors [28], practical hydrogen sensors [29], and gas sensor design [30] have been reviewed by some researchers. The semiconductor gas sensor is not an energy conversion but energy control type sensor. The physical properties of a sensing material change upon exposure to gas molecules, and external electric energy transmits the change as a sensor signal. This implies that, in most cases, the electrode of the semiconductor gas sensor is similar to that of an electronic device, which delivers current flow or electric power supply without loss or supplies electric energy from external power sources to the device. Thus, in conventional electronic devices, the electrode only connects the device and external circuit. Accordingly, a strong mechanical adhesion and small contact resistance are the most significant factors; in addition, durability, chemical resistance, reliability, and cost should be considered [31,32,33,34,35]. However, the electrode of a semiconductor gas sensor not only measures the electric properties of the sensor but also measures the catalytic properties of the sensing material. The Ohmic electric contact made between the device and the electrode material is acceptable; however, the semiconductor gas sensor sometimes requires a rectifying contact between the sensing material and electrode. A rectifying contact would create a dipole in the interfacial zone of a metal and semiconductor triggered by gas adsorption, reducing a potential barrier from time to time or leading to complex phenomena such as field emission or tunneling effect due to thermionic field emission [36,37,38,39,40,41,42]. In special cases where the semiconductor gas sensor is applied to cars or in the aerospace industry, the electrode material should be able to operate above 600 °C [5,43].

This study is aimed to review the impact of the electrode used by the semiconductor gas sensor on the operation of a gas sensor. In this paper, the electrode types of the semiconductor gas sensor that have been made available so far are discussed and the electrode materials for the gas sensor are presented. The carrier transport mechanism in electrode–semiconductor interfaces and effects of gas adsorption on the electrode in the semiconductor gas sensor are discussed.

## 2. Electrode Types of Semiconductor Gas Sensors

The electrodes used for gas sensors should be in contact with the substrates, and their electrical properties should be easily measured. The following conditions are thus required:

(1) They should be chemically and mechanically stable on the substrates.
(2) The connection to the lead-out terminals should be easy.
(3) The sensing film should not be damaged during electrode formation.
(4) They must have a geometry that is suitable for sensor construction.

### 2.1. Two-Electrode Configuration

**Figure 1:**
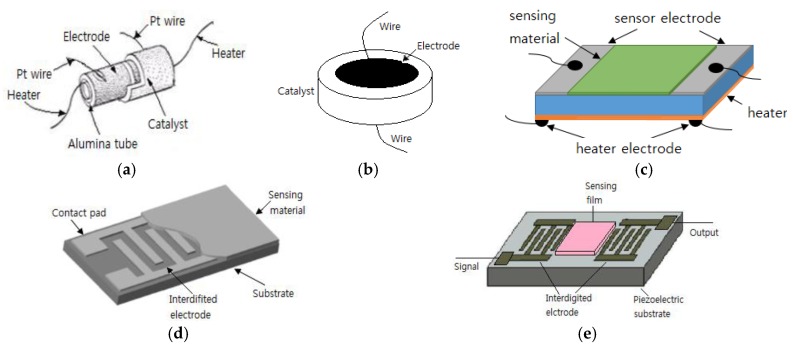
Two electrode configuration used with gas sensors; (**a**) cylinder; (**b**) disk; (**c**) parallel plates; (**d**) interdigit and (**e**) surface acoustic wave (SAW) line.

The two-electrode type configuration, in which the gas sensing material is positioned between two metal electrodes, is the most widely applied one to semiconductor gas sensors. Occasionally, a third electrode is used as a heater for the sensors. Toohey [44] explained the electrode types used in a semiconductor gas sensor. Two-electrode configurations are used for gas sensors, as shown in Figure 1. In type (a), the Pt electrode is formed on an alumina cylinder, which is applied to a Figaro sensor, and then the sensing materials are deposited on it and sintered. In type (b), a tablet made of an oxide semiconductor is sintered and then the electrode is formed on both sides. In type (c), two combs face each other to create an interdigitated geometry on the substrate. The transmission type (d) sensor is formed in order to fabricate a surface acoustic wave (SAW) filter that measures the frequency changes. The interdigitated geometry is the most widely accepted geometry for the electrodes of a gas sensor since it enables a wide contact area between the electrodes within the limited area. In addition, it forms the electrodes first and then deposits the sensing materials on them, thereby causing no damage to the sensing materials.

### 2.2. One-Electrode Configuration

**Figure 2:**
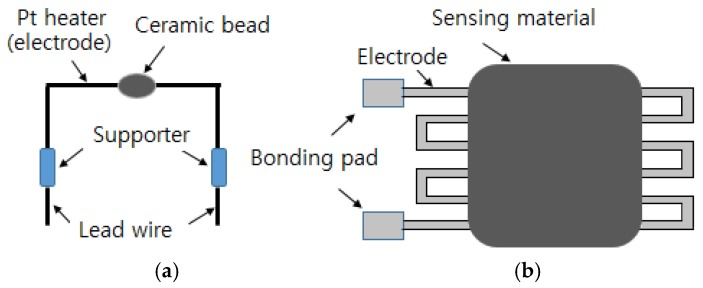
One-electrode configuration; (**a**) ceramic bead surrounding Pt electrode and (**b**) Pd electrode on alumina substrate.

A one-electrode configuration that differs from the two-electrode type that has been previously used for semiconductor gas sensors has been developed. Korotcenkov [31] reviewed the design and type of the one-electrode configuration for semiconductor gas sensors. One electrode acts as both the heater and the measuring terminal, unlike the two electrode setup. As demonstrated in Figure 2, one-electrode gas sensors can be formed by applying the metal oxide in the form of a bead on the electrode material or by shunting the electrode through a coating [31]. Materials such as SnO_2_ [45,46,47,48], In_2_O_3_ [49], and Fe_2_O_3_ [50,51] are mainly used for one-electrode gas sensors.

For operation of the one-electrode sensors, impedance matching should be performed between the shunting semiconductor resistance and the electrode resistance, as shown in Figure 2. One can adjust the electrode resistance by varying the electrode thickness, distance between the electrodes, or resistance of the sensing materials by adding additives to the oxide semiconductor or by modifying the thickness [31].

Faglia et al. [52] used four electrodes in order to analyze whether the contact and the grain is more important for gas detection. Their study revealed that the contact is more important for CO gas detection, whereas the material used is more important for CH_4_ gas detection. Four electrodes have been applied to the sensor which used CrTiO_3_ [53] and WO_3_/TiO_2_ [54] as sensing materials.

This implies that the material and geometric structure of the electrodes significantly influence the operation of gas sensors. Many researchers studying nanosized sensing materials perform studies on both microsized electrodes and nanoelectrodes with a single particle size of approximately 5 nm. The advantages of nanoelectrodes are as follows: [44] (i) the ability to arrange in single nanodots; (ii) the ability to vary the relative contributions of electrode–dot and dot–dot contacts to the total sensor resistance; (iii) where a nanodot film consists of conducting and non-conducting particles, decreasing the electrode size could increase sensitivity by around an order of magnitude or more by “softening” the percolation threshold [55]; and (iv) the ability to reduce the chip area due to the small size of the electrode.

### 2.3. Gate Electrode

As all electronic parts become integrated and intelligent, it is also inevitable to make small and integrated gas sensors. So far, many researchers have used conventional metal-insulator-semiconductor field effect transistors (MISFETs) [56,57,58,59,60,61,62,63,64,65,66] or micro-electro-mechanical systems (MEMSs) [67,68,69,70,71,72,73] to manufacture semiconductor gas sensors. Gas detection with such technologies depends on the varying conductivity owing to gas adsorption and the reaction on the MIS structure surface or varying work functions of the MISFET resulting from catalytic reaction in the gate electrode. However, sensor stability is not ensured yet though MIS gas sensors are increasingly needed. Since the gate electrode is exposed, unintended reactions between the gate electrode and materials near it reduce the sensor sensitivity or selectivity with time and it takes longer to respond to the gas molecules.

**Figure 3:**
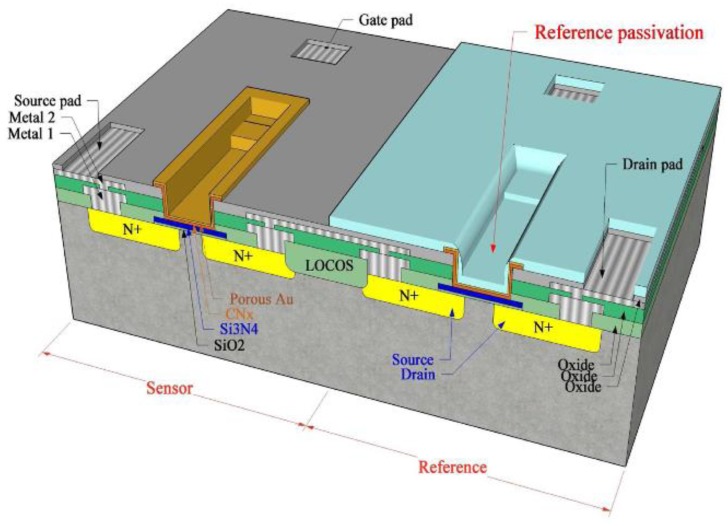
Design of differential humidity sensitive field effect transistors with porous Au gate.

The gate electrode of the CO gas sensors with the MIS structure needs to apply a voltage for the device so as to form a channel and also carry out catalytic actions [66]. Thus, the electrode can be made porous so that the area where the adsorbed gases contact the sensing materials increases instead of the gate covering the surface of the sensing materials. Janata et al. [74] created a suspended microgrid on a FET gate to extend the lifetime of the sensing gas. In these devices, the gate metal is preceded by an additional space, which, in the case of GasFET, is permeable to gases. The suspended grid above the gate insulator is made of Pt or Au. Applying a Pd layer to this creates a hydrogen sensor. If a conductive polymer layer, such as polypyrrole, is deposited on the metal grid, then the sensor is sensitive to alcohols. In both cases, the reaction of the gas with the surface of the suspended metal grid or with the surface of the insulator causes a change in the electric field that is detected in the modified drain current [75]. Lee et al. [16,76] have tried to deposit a porous metal gate for humidity sensitive field effect transistors (HUSFETs) that can sense humidity. Here, a thin gold film of approximately 100 Å through which water molecules could penetrate was deposited on the active layer before a pattern was formed using lift-off techniques. When water molecules meet carbon nitride through the porous gold layer of the gate in Figure 3, adsorbed water molecules on the carbon nitride are able to form dipole and to reorient freely under an applied gate voltage, resulting in an increase in the dielectric constant [16]. Thereafter, Fukuda et al. [66] applied porous Pt as gate electrode materials to improve the sensitivity of the MOSFET-type hydrogen sensors. When a porous electrode was used, the sensor detected 22 ppm of H_2_ gas in less than 2 min, thus indicating a remarkable gas detecting performance. Its sensitivity level was enhanced by approximately ten times as compared to that of a non-porous Pt surface because of the catalytic property of the porous Pt surface. For the purpose of detection of negative ions in the air, Lee et al. [77] have suggested a nanoFET sensor that uses a Ti-Al layer as the electrode for the source and drain, while using a floated Ti/Au layer as the electrode on the gate oxide.

### 2.4. Electrode Geometry

Many researchers have studied the influence of the geometry and position of electrodes on the sensitivity and selectivity of sensors [44,78,79,80,81,82,83,84]. The width of digits in interdigitated electrodes or the space between the electrodes can affect the sensor performance. In other words, when the electrode spacing is narrow, the current between electrodes flows only in the film area right above it. On the contrary, when the spacing is wide, the current flows both horizontally and vertically throughout the film, thereby sampling a wider area [44,79]. In addition, the electrode-semiconductor interface itself can cause a change in the device sensitive resistance. When the width/gap ratio of the electrode is changed, the influence of both the interface and the film resistance on sensitivity can be relatively reduced. Vilanova et al. [80] investigated the effects of the electrode position, electrode gap, and active layer thickness on sensor/gas pairs with high-level, medium-level, and poor-level catalytic activity.

Gases diffuse by spreading through the film reacting with the particle surface, thus resulting in a local change in the film resistance. It has been observed through simulations that there is an increase in the sensitivity following an increase in the spacing between the electrodes when the electrodes were placed under a sensing film if the sensor was very sensitive to the gas. In contrast, when an electrode was placed above the sensing film, the sensor sensitivity decrease as the spacing between the electrodes increased. The result of injecting a highly reactive gas was same as the result of injecting a low reactive gas when the gap between the electrodes decreased and became smaller than the film’s thickness. The sensitivity depends on electrode spacing for a sensor whose electrode is placed below the sensor film. In this case, the detection level for even a highly reactive gas was observed to be the same as that of a low-reactive gas. On the contrary, when the electrode gap is sufficiently wide, the detection level of even a low-reactive gas was observed to be the same as that of a highly reactive gas. Therefore, the gas detection performance of a sensor with an electrode placed below its film is better when the electrode width is wide and electrode spacing is narrow [81].

Tamaki et al. studied the effects of gap size differences between electrodes [82]. Microgap electrodes (0.1–1.5 μm) were formed on the silicon substrate using the MEMS process, and WO_3_ films were deposited on these electrodes. When the gap size was larger than 0.8 μm, there was no change in sensitivity to NO_2_, however, when it was smaller than 0.8 μm, the sensitivity tended to increase and was expected to increase further in the range of less than 0.1 μm. They explained that this is caused by the number of grains of WO_3_ in the microgap and the resistance change at the boundaries. Saalan et al. [83] also fabricated microgap electrodes (1–30 μm) by dc-sputtering and FIB techniques and deposited SnO_2_ nanowires on them using the suspension dropping method. They suggested that the interface between the electrodes and the sensing area play a very important role in the sensing mechanism of SnO_2_ gas sensors. Comparison between the small gap and large gap electrodes showed that the small gap electrode had the advantage of reliability and high sensitivity to low NO_2_ concentration, whereas the large gap electrode had relatively high sensitivity for high concentrations. Hoefer et al. [84,85] analyzed the influences of the electrode width and gap on contact resistance in tin oxide sensors by using the transmission line method. For CO gas sensors, sensitivity is the highest when the SnO_2_ layer is wide and the electrode gap is narrow. The sensitivity was low when the SnO_2_ layer was narrow. In contrast, for NO_2_ gas sensors, sensitivity was decreased following an increase in the width of the layer.

## 3. Electrode Materials for Semiconductor Gas Sensors

Many researchers have long studied the interaction between the electrode and sensor materials as well as the impact of the electrode materials on the sensing behavior [86,87,88,89,90,91,92,93,94,95,96,97,98,99]. The types of electrode materials used for semiconductor gas sensors are classified into bulk, thick film, and thin film. The bulk type is rarely used for the semiconductor gas sensor. The thick film type, which performs screen printing by producing a paste, and the thin film type by vacuum deposition are employed in many cases.

The impact of the electrode on the properties of gas sensors based on tin oxide has been studied mainly by comparing various electrode materials such as Au, Pt, and Pd [36,86,87,88]. Capone et al. [86] analyzed the impacts of two different interdigitated electrode geometries on the sensitivity of two different electrode materials (Au and Pt) for CO gas. These studies revealed that the Au electrode had a lower stability level than the Pt electrode. With regard to temperature, the sensitivity of CO was the highest at approximately 300 °C for the Au electrode and at 450 °C for Pt electrode, and it was observed to decrease slightly at low temperatures. In addition, a pure tin oxide sensor has displayed linear current-voltage properties under all conditions, whereas a sensor with an additive has shown nonlinear properties. The Pd and Au electrodes had nonlinear characteristics, but the Pt electrode had linear characteristics for high hydrogen concentrations. These studies reported that the electrode-semiconductor contact exerts substantial influence on the entire sensor impedance. Both a device made of tin oxide and a device made of 10:1 mixture of tin oxide and manganese oxide have shown the highest level of sensitivity in a temperature range of approximately 350 °C–450 °C [87]. When SnO_2_ thick film gas sensors that use Au and Pt as electrode materials were tested for hydrogen and CO gas, it was observed that the Pt electrode was more sensitive to H_2_, whereas the Au electrode was more sensitive to CO [88]. Durrani [89] used Ag, Al, Au, and Pt to study the effect of electrode material on the SnO_2_-based CO thin film gas sensor. Pt and Au showed higher response than Ag or Al when the electrode material was below the sensing material. In addition, Gourari et al. [87], Pijolat [90], and Bertrand et al. [91] have studied Pt and Au as electrode materials in SnO_2_ gas sensors.

Schottky-type sensors, in which the metal and semiconductor are in contact, are most widely used as hydrogen sensors. When the gas is not adsorbed in Schottky-type sensors, the energy band of the semiconductor bends upwards or downwards by the difference in the Fermi level between the metal and the semiconductor in the thermal equilibrium state. Such a situation arises when there is a thin insulator layer between the metal and semiconductor as well [30,35]. In general, Pd is used as the electrode material for Schottky-type H_2_ sensors. When hydrogen molecules are adsorbed onto Pd, which is a catalytic metal, they disassociate to become hydrogen ions. Some of these ions permeate Pd, spread toward the metal-semiconductor interface, form dipoles, and then change the metal’s work function and consequently, its barrier height. A change in the barrier height changes the current-voltage properties and this amount of change determines the detection of hydrogen concentration.

In MISFET-type hydrogen sensors, the threshold voltage in the gate layer changes based on the hydrogen concentration, resulting in a change in the drain current. Hydrogen sensors that use Schottky diodes were proposed for the first time by Lundstrom et al. [56] and Steele et al. [99]. For both studies, Pd was used as the electrode and the semiconductor substrate used was n-Si and CdS, respectively. In 1979, Ito [100] produced Schottky sensors that used SnO_2_, In_2_O_3_, KTaO_3_, ZnO, and Pd as the electrode and stated that they reacted to hydrogen. In a study comparing Schottky diodes that used Pd and Pt as the catalytic metals, it was observed that the one with Pt showed higher sensitivity than the one with Pd [101,102]. In addition to Pd and Pt, hydrogen sensors that use pure metals or alloys such as Ru [103], Ni [104], Au [105], Ag [106], IrPt, and PdAg [107] were also suggested. Song et al. [107] fabricated Schottky diodes by using AlGaN-GaN, as well as metal materials such as Pt, IrPt, and PdAg, in order to investigate their reaction to hydrogen gas. In a relatively low temperature range of 200 °C–300 °C, PdAg showed higher sensitivity, whereas above 400 °C, IrPt and Pt showed higher sensitivity. A diode that uses the PdAg material displayed poor thermal stability. Studies on the electrode effects of semiconductor gas sensors are summarized in Table 1.

The drawback of gas sensors that use oxide semiconductors is that their reliability declines with time. Drift occurs in the sensor properties and the long-term stability is diminished. Important factors in selecting an electrode material for a gas sensor include long-term stability, heat resistance, chemical resistance, and adhesion to a substrate. Long-term investigations are seen as an important criterion that determines the usability of the sensors. Meixner et al. [108] reported that the main reasons for inadequate long-term stability are the change of the metal oxide, the change of the metal electrode, instability of the wire contacts, and interaction with an unsuitable sensor casing.

The degradation of contacts takes place mainly due to the diffusion occurring at the electrode and oxide interface or the interaction of electrode with the surrounding atmosphere [109]. Among the electrode materials for semiconductor gas sensors, Ag is stable in air and used over a wide temperature range. However, Ag has a low long-term stability disadvantage and the degradation of contacts. Ag can easily move or migrate at temperatures above 300 °C. Au is also one of the most popular electrode materials owing to its high electric conductivity and reliability. However, it has the disadvantage of easily diffusing into the substrate (especially silicon) at a relatively low temperature. On the other hand, Pt is the most stable electrode material, with little degradation. However, it is expensive and has poor substrate adhesion. In order to improve the adhesion to the substrate, a glue layer of Cr, Ti, or W is formed between the electrode and the substrate. For good adhesion, Hoefer et al. [110] used Ta, whereas Michel et al. [111] used TiN as the glue layer. Sozza et al. [112] also reported that the Ti/Pt layer can prevent the rather fast degradation as compared to the Ti/Au layer or the Ti/Pd/Au layer. Capone et al. [113] studied the influence of electrode aging on pure SnO_2_ thin films, and in the SnO_2_ films with Ni, Os, Pt, and Pd as an additives. The electrode configuration was interdigitated. The drift was lower for the cases in which Ti/Pt were used as the electrode materials than that wherein Ti/Au or Ti/Pd/Au were used.

**Figure 4:**
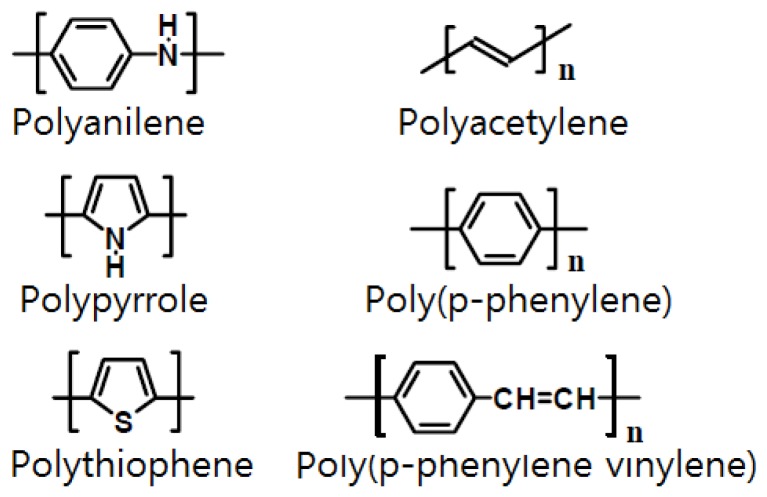
Various conductive polymers.

**Figure 5:**
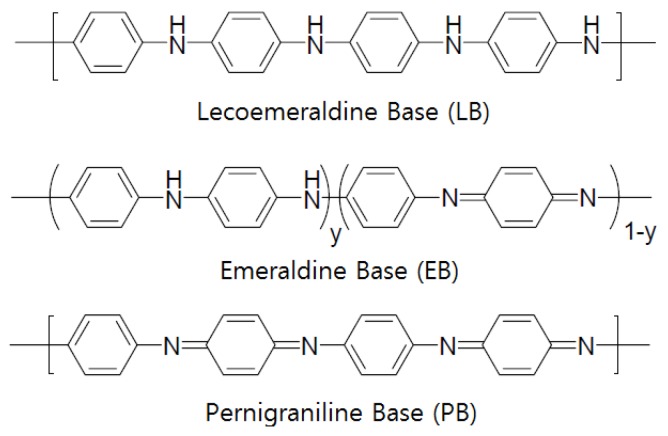
Polyaniline bases according to oxidation states.

Some semiconductor gas sensors use a conductive polymer as the electrode material. Most organic polymers are electrically non-conductive, but conductive polymers can be produced by providing a channel for electrons to travel along polymer chains or to jump from chain to chain [114]. Such conductive polymers include polyaniline, polyacetylene, polypyrrole, poly(*p*-phenylene), polythiophene, and poly(*p*-phenylenevinylene), among which polyaniline (PANI) is the most widely used [115,116,117]. Figure 4 shows examples of conductive polymers. Polyaniline has received significant attention as it has a high electrical conductance of 10^3^ S/cm and has been reported to have metallic properties. According to the synthesis method, polyaniline can be divided into the following states (Figure 5): (i) completely oxidized state (PB: 1−y=0, quinoid); (ii) intermediate oxidation state (EB: 1−y=0.5); and (iii) completely deoxidized state (LB: 1−y=1, benznoid). EB is generally easily produced using an oxidizer such as (NH_4_)_2_S_2_O_8_ to oxidize aniline directly in the presence of a protonic acid. LB can be easily obtained by applying a reducing agent such as hydrazine hydrate to EB, whereas PB can be produced using an oxidant such as *m*-chloroperoxybenzoic acid [118,119].

Conductive polymers have a high conductance of approximately 10^3^ S/cm as compared to that of an ITO electrode. Thus, they can enable the production of thin films through spin coating, which is much more economical and convenient than evaporation or sputtering. However, conductive polymers have some disadvantages, such as a property change during the production of doped polymer composition, the use of a non-volatile solvent (*m*-cresol), and their color. For realizing a flexible sensor system in future, the metal electrode materials must be replaced by organic materials. Among organic materials, monomolecular pentacene has the highest level of charge transfer [115]. Pentacene, however, has a disadvantage in terms of its manufacturing process, such as it is impossible to effect vacuum evaporation. Polythiophene derivatives are used as conductive polymers to replace pentacene, and they have high electric field mobility, however, they show a relatively low on-and-off ratio. Polyaniline and polypyrrole also have a low on-and-off ratio, but the ratio can be enhanced since the conductivity level of nanostructured polyaniline can be more easily adjusted than the doping level as compared to polymers. The replacement of the electrode material, which is the core part of flexible devices, is very important. Many experts expect that when the replacement technology is accomplished, the development of flexible devices will reach the stage of completion. Polyaniline can also be applied to a variety of fields, such as the electrode material of sensors, insulation layer of O-TFT, and channel material of an electrical transport layer. Notably, for polyaniline, the electrical conduction of metals and semiconductors can be much more easily controlled by adjusting the protonic acid doping levels and by using appropriate methods than for carbon nanotubes [115].

Recently, the field of printed electronics has been receiving considerable attention due to the development of semiconductor fabrication technology. It is important for semiconductor gas sensors to make electrodes by printing the materials. The most commonly used materials for electrodes are novel metals, such as silver, gold, platinum, and palladium. The alloys of these metals are also widely used. Table 2 summarizes the properties of metal inks for semiconductor gas sensors [120].

## 4. Interfacial Zones of Electrode-Semiconductor Contact

A barrier of metal/semiconductor contact is a limiting situation which can be described as two infinite half planes of material, one a metal and the other a semiconductor, brought into contact. A typical technical barrier would result from contacting metal on the semiconductor after a series of in-ambient preparations. An ideal barrier results from depositing the metal in a carefully controlled way where precautions are taken to keep the interface atomically clean, that is, the only atoms present are those of the initial semiconductor and the desired metal. The procedure for cleaning the surface may strongly influence the outcome by altering the surface structure stoichiometry or by introducing surface defects. Two regimes are:

(a) Building up the metal layer by layer by evaporation deposition
(b) Pressing two bulk pieces together to form a point contact

**Figure 6:**
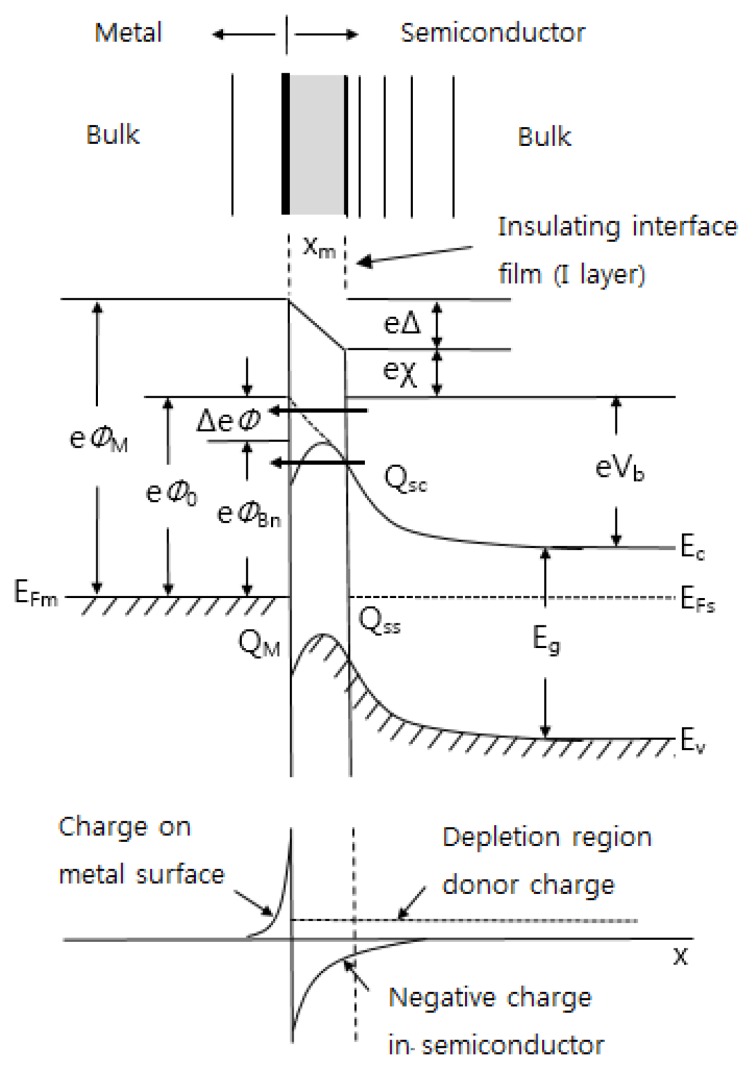
Schematic depiction of interfacial zones on the Schottky junction. *Φ_M_* is the work function of metal, *Φ_Bn_* is the barrier height of metal-semiconductor barrier, *Φ_0_* is an asymptotic value of *Φ_Bn_* at zero electric field, Δ*Φ* is image force lowering, Δ is potential across interfacial layer, χ is electron affinity of semiconductor, *V_b_* is built-in potential, *Q_sc_* is the space charge density in semiconductor, and *Q_M_* is the space charge density on metal, respectively.

In both cases, the technical interface would have amount of extraneous material trapped at the interface. In actual situations, this material might consist of a native oxide of 10–15 Å [33]. In metal-semiconductor contacts, the interface layer has three zones as depicted schematically in Figure 6 [121,122,123,124,125,126,127]. On the metal side, the alloyed zone may be of sufficient width to become the metal forming the barrier. There is an insulating layer present, either intentionally or unintentionally, between the metal and the semiconductor. This layer can be a purposefully deposited layer or a thermally grown layer. The layer could also inadvertently result from the act of junction formation. There can be an interfacial or transition layer extending into the semiconductor due to out-diffusion of constituents of the semiconductor, in-diffusion of metal atoms, chemical interactions, and semiconductor surface damage. The presence of insulating layer can have two effects on barrier formation: one is a geometrical effect (it can simply further separate the charge in the metal from the charge in the semiconductor, giving a large dipole length), and the other is a potentially significant modification of the dipole arrangement. If the interfacial layer contains no charge, then its effect on barrier formation would be geometrical. However, the presence of an I layer together with interface states has more than a geometrical effect. It can strongly modify the dipole arrangement since these localized states can hold charge. Such interface states can be extrinsic arising from defects caused by cross diffusion, chemical interaction, and semiconductor surface damage and rearrangement. Interface states can also be intrinsic: they can be a basic feature of the semiconductor surface or they can arise from the extension of metal electron wave function into the semiconductor gap. Since interface states can store charge, they can modify the field in the I layer. It is seen that their presence, together with the I layer, can modify *V_b_* and *Φ_B_*. Unlike the simple geometrical case, it is now possible to increase *V_b_* and *Φ_B_* or decrease them, depending on the charge stored in the interface states [121].

On the semiconductor side, the interface states are distributed within the two zones, one being derived from the evanescent tail of the metal wave function into the semiconductor and the remaining interface layer being the second zone. The interface states in zone 1 in many cases have the most significant effect on the determining the barrier height. The first zone is at most 10 Å thick from estimates available in literature [121,122]. The second zone can be very large depending on the thermal history of the sample and can also significantly affect the applied bias performance of the barriers.

When a metal is contacted with a semiconductor, the contact area formation, energy band diagram, and charge distribution on the Schottky junction are shown in Figure 6. As mentioned above, when analyzing the bonding between metal and semiconductor, it is necessary to consider a thin insulating layer at the interface.This is because the insulating layer decouples the metal from the semiconductor so that each of them can be treated as a separate system. One can then regard the interface states as a property of the particular semiconductor-insulator combination and ignore any modification in the surface dipole contributions to the work functions of the metal and the semiconductor. These simplifications obviously are not possible in the case of clean contacts [108]. If there is no insulating film in the metal-semiconductor contact, the effect of lowering the barrier by the image force appears. This is called the image force lowering. If an electron is at a distance *x* from the metal surface, then there exists an electric field perpendicular to the metal surface. This is like the effect of a positive charge at a distance *-x* inside of a metal. It is seen from Figure 6 that the maximum in energy occurs at a distance *x_m_* from the metal surface and it can be shown that the magnitude Δ*Ф* of the barrier lowering is given by [33]:

(1) $$\Delta e\varphi ={\left[\frac{e^{3}N_{d}}{8\pi^{2}\epsilon_{d}^{2}\epsilon_{s}}\left(V_{b}-V\right)\right]}^{1/4}$$

where *N_d_* is the donor concentration, and *V* is the applied voltage.

Figure 6 can in fact be used to characterize any of the metal-semiconductor contacts such as semiconductor-semiconductor and metal-insulator-semiconductor. Using this approach, the difference in these cases depends on how much freedom exists in specifying the electric field at the interface. The various interface formation regimes are listed in Table 3 [33].

Metal/semiconductor contacts are fabricated with the layer-by layer approach, or the deposition of the metal on the semiconductor. Following the preparation of the clean surface, the initial state of the semiconductor surface can have many different configuration which will affect the outcome of the junction barrier that forms. The state can consist of either structural or compositional disorder reflected in structural defects such as steps, surface vacancies, or anti-site arrangement.

In the initial metal deposition, the density of surface atoms, which can be determined accurately, is low enough that they do not interact. In this case, one can identify site-specific chemisorption as well as reaction or replacement. Energy released at this stage such as the heat of condensation or reaction may dislodge other surface atoms from their equilibrium sites. The outcome of this stage may depend upon the surface temperature either through surface diffusion or through activated processes. As the surface concentration grows, the possibility of metal-metal interactions become a significant aspect. Depending upon the surface mobility and strength of the interaction, the metal may be have a tendency to form clusters. For many metals, the cluster formation or metal film nucleation is exothermic, so this stage can disrupt the underlying lattice. Beyond the development of the initial metal overlayer, depending on the configuration of the metal the metal film may be under a significant stress (either tensile or compressional), which can be a driving force for interdiffusion and at some point dislocation formation. Stress, dipole formation, and other phenomema drive interdiffusion which can proceed over tens of angstroms even at room temperature. The final interface may be compositionally and spatially inhomogeneous so that there is significant dimension to the situations that need to be evaluated detail. These effects can be accentuated with the thermal processing that usually accompanies device processing [128,129,130].

One can choose a metal to fabricate Schottky barrier sensors with a particular semiconductor sensing material. A key of this choice is metal and semiconductor work functions. For an n-type semiconductor, the metal work function *φ_M_* should be greater than the electron affinity *x* of the semiconductor, while for a p-type semiconductor, it should be opposite. The barrier height in Figure 6 is given by:

(2) $$\varphi_{Bn}=\varphi_{M}-x$$

For an ideal case, the barrier height is about equal to the band gap of the semiconductor. In practical, however, the interface between metal and semiconductor becomes a very complicated state due to due to interface states originating either from surface states [121] or from metal-induced gap states [125] and/or due to interface chemical reactions of metal and semiconductor atoms [131,132,133]. We need to modify the Equation (2). Cowley and Sze [125] have derived an expression for *φ_Bn_* taking surface states into account. Then one obtains:

(3) $$\varphi_{Bn}=\gamma \left(\varphi_{M}-\chi \right)+\left(1-\gamma \right)\left(E_{g}-\varphi_{0}\right)-\Delta \varphi$$

And:

(4) $$\gamma =\epsilon_{i}\left(\epsilon +e^{2}x_{m}D_{s}\right)$$

where *x_m_* is the thickness of the interfacial layer, and *D_s_* the density of interface states. Neglecting Δ*Ф*, Equation (3) reduces to Equation (2) when *Ds* = 0. In the literature, there are numerous experimental date [33,134,135,136] on *Ф_M_*. Table 4 gives the most preferred experimental values of work function for important metals.

The other important factor on which metal selection depends include its low diffusivity in the semiconductor and its ease of deposition (convenient temperature of deposition and good adhesion), no interface reaction, good electrical and thermal behavior, and adaptability to thermocompression bonding. The reactivity of a metal with the semiconductor is one the most important factors affecting Schottky barriers. It is, therefore, to know the reactive or nonreactive metals with regard to a particular semiconductor prior to selecting an electrode metal for sensors.

For a heavily doped semiconductor, the energy barrier is sufficiently thin for electrons to tunnel through the barrier, resulting in an ohmic contact. Tunneling theory has been analyzed by Padovani and Stratton [137] and by Crowell and Rideout [138]. The current-voltage characteristics in the presence of tunneling can be modified by:

(5) $$I=I_{s}exp\left(\frac{eV}{E_{t}}\right)$$

where:

(6) $$E_{t}=E_{00}coth\left(\frac{E_{t0}}{kT}\right)$$

**Figure 7:**
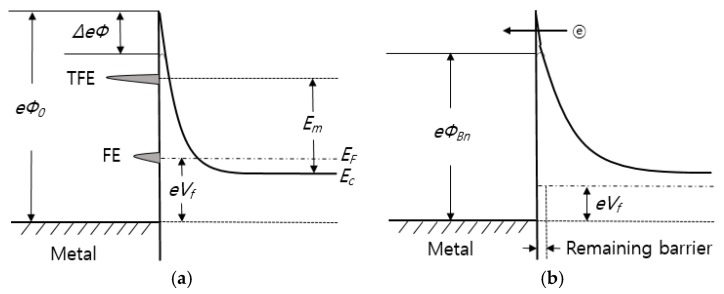
Energy band diagram of metal-semiconductor under forward bias: (**a**) highly doped and (**b**) lightly doped.

And:

(7) $$E_{t0}=\frac{eh}{4\pi }{\left(\frac{N_{d}}{m^{*}\epsilon_{s}}\right)}^{1/2}$$

where m* is the electron effective mass and *h* is Planck’s constant. Contribution of thermionic field emission (TFE) to the diode current dominates for *E_00_~kT*. The energy *E_m_* at which TFE has its maximum contribution occurs at:

(8) $$E_{m}=\frac{eV_{b}}{{\left[cosh\left(E_{00}/kT\right)\right]}^{2}}$$

where *V_b_* is the voltage corresponding to the total band bending and *E_m_* is measured from bottom of the conduction band at the edge of the depletion region. The analysis of them [137] on which the above results are based has neglected the image force barrier lowering and quantum mechanical reflections of electrons from the top of the barrier. Moreover, in both the above works the electron distribution was assumed to be described by Boltzman statistics. On the other hand, studies on tunneling effects in lightly doped semiconductors have also been reported in the absence of the interface insulating layer. An explanation for this ohmic behavior to lightly doped material is the formation of semiconductor vacancies during alloying with subsequent metal atoms occupying semiconductor sites and becoming donors. For the case where a low barrier results, it is supposed that a thin layer (10 Å) of degenerate material exists at the surface that modified the barrier shape as shown in Figure 7 [137]. A low forward bias is required to raise the majority carrier over the first part of the barrier by thermionic emission but the remaining barrier is sufficiently thin for tunneling to occur.

The understanding of the mechanism of barrier formation at the metal-semiconductor contact is still far from complete although considerable progress in understanding the physical processes occurring at the interface has been made during the decades. Ideal conditions as postulated by Schottky and Bardeen [139,140] hardly ever exist at the interface, and the dipole layer depends upon the spatial arrangement of constituent atoms at the interface and whether or not they form chemical bonds with each other. Thus, the type of interface appears to play an important role in the formation of the barrier. For reactive interfaces where the metal forms compounds with the semiconductor the barrier height may be correlated with parameters related to the metallurgical reaction such as heat of formation or the eutectic temperature of the interfacial compounds. Other type of contacts most frequently encountered in sensors are those in which there is a thin oxide layer between the metal and the semiconductor as shown in Figure 6. In these contacts the barrier height depends upon the method of preparation of semiconductor surface and tends to increase with increasing value of the metal work function although the relation between *φ_Bn_* and *φ_M_* is not strictly linear. The presence of oxide produces interface states which depends only on the oxide semiconductor combination and the modification of these states by the presence the metal is prevented to a large extent. The perturbing action of the metal comes through the penetration of the metal electron wave functions into the semiconductor forbidden gap and by interdiffusion of atoms across the boundary.

## 5. Conclusions

Identifying the phenomena that occur between the electrode and semiconductor of the semiconductor gas sensor has been a very interesting topic for decades. The types or application methods of the semiconductor gas sensor determine the electrode type. One electrode sensors using micromachining technology are widely applied, replacing the traditional interdigit two electrode type. As a planar design is applied to the one electrode sensor that is produced with the micromachining technology, large scale integration is appropriate. In addition, it has advantages of low power consumption and production cost. For the microsensor represented as the FET type, materials used for the gate electrode and the type of configuration are essential. When it works simply as a device that supplies voltage to the gate, poly Si or aluminum can be used. When the gate electrode should perform a catalytic function or allow gas penetration, special materials are used or a unique design is applied. Many studies also attempt to apply conductive polymer to the gas sensor. The conductive polymer electrode using polypyrrole (PPy), and polyaniline (Pani) is manufactured with casting, layer-by-layer deposition, spin-coating or LB techniques. Its manufacturing process is simple which allows room temperature operation. However, it is challenging to apply the techniques to the high temperature FET type because the semiconductor fabrication process is carried out at a temperature higher than 1000 °C. It appears that the charge transport theory can be analyzed easily with the Shottky-Mott model for the electrode-semiconductor interface. However, different analyses are possible depending on the sensor type and many studies failed to reach a clear conclusion. This implies that one single mechanism cannot explain different material situations.

## Figures and Tables

**Table 1:** Studies for electrode effects of semiconductor gas sensors.

Electrode Materials	Sensing Materials	Target Gases	References
Pd, Pt	ZnO, SnO_2_, In_2_O_3_, KTaO_3_	H_2_	Ito [100]
Au, Pd, Pt	SnO_2_	H_2_	Gourari [87]
Ru	SiC	H_2_	Basu et al. [103]
Pt, IrPt, PdAg	Al GaN-GaN	H_2_	Song et al. [107]
Ni	Si	H_2_	Salehi et al. [104]
Au	ZnO	H_2_	Pandis et al. [105]
Au, Pt	SnO_2_	H_2_, CO	Rank et al. [95]
Au, Pt	SnO_2_	H_2_, CO	Saukko et al. [88]
Au, Pt	SnO_2_	CO	Capone et al. [86]
Au, Pt	SnO_2_	CO	Bertland et al. [91]
Ag, Al, Au, Pt	SnO_2_	CO	Durrani [89]
Au	Fe_2_O_3_-In_2_O_3_	CO	Golovanov et al. [51]
Ag, Au	ZnO	CO, NO_2_	Lin et al. [93]
Au	WO_3_	NO_2_	Tamaki et al. [82]
Au	SnO_2_	NO_2_	Shaalan et al. [83]
Au, Pt	SnO_2_	Benzene	Pijolat [90]
Pt, Au, Pt-Au	SnO_2_	H_2_O	Ylinampa et al. [92]
Al	WO_3_	Cl_2_	Bender et al. [94]

**Table 2:** Properties of printing metals and alloys for electrode of semiconductor gas sensors.

Materials	Electrical Properties	Advantages	Disadvantages
Silver	- High conductivity - Compatible with resistor and dielectric system - Resistivity: 1.59 × 10^−8^ Ω m	- Least expensive - Good bond strength	- Tendency to migrate over the surface of insulants and resistors under high humidity
Gold	- High conductivity and reliability - Resistivity: 2.44 × 10^−8^ Ω m	- Alloy with tin may be made without the use of flux	- High cost - Unsuitability for solder joining
Platinum	- Use where extreme resistance to molten solder and to bond strength degradation by solder is required - Resistivity: 11.0 × 10^−8^ Ω m	- Available wire, flat plate, and tube - Large range of size - Usable at high temperature	- Most expensive
Palladium-Silver	- Compatible with resistor and dielectric system - Sheet resistance: 0.01–0.04 Ω/sq	- Suitable for ultrasonic wire bonding	- The possibility of silver migration under high humidity
Platinum-Silver	- Alternative to Pd-Ag - Sheet resistance: 0.01–0.04 Ω/sq		- Not recommended for hybrid applications involving ultrasonic wire bonding
Platinum-Gold	- Compatible with most thick film materials - Sheet resistance: 0.08–0.1 Ω/sq	- Excellent solderability - Suitable for both wire and die bonding	- High cost - Rather high electrical resistivity
Palladium-Gold	- Similar properties to Pt-Au - Sheet resistance: 0.04–0.10 Ω/sq	- Less expensive than Pt-Au	- Inferior solder leach resistance and solder ageing than Pt-Au

**Table 3:** Interface formation regimes.

a) Metal deposition onto cleaved surfaces b) Metal deposition onto sputtered surfaces c) Metal deposition onto sputtered and reannealed surfaces d) Single crystal metal growth on in situ grown semiconductors

**Table 4:** Work function of some important metals.

Metal	Work Function (eV)	Metal	Work Function (eV)
Pt	5.65	Zn	4.33
Ni	5.25	Al	4.28
Pd	5.12	Ag	4.26
Au	5.1	Pb	4.25
Cu	4.65	Ta	4.25
W	4.55	Cd	4.22
Cr	4.5	Ga	4.2
Hg	4.49	In	4.12
Sn	4.42	Zr	4.05
Ti	4.33	Cs	2.14
